# Selective Preparation of *trans*-Carveol over Ceria Supported Mesoporous Materials MCM-41 and SBA-15

**DOI:** 10.3390/ma6052103

**Published:** 2013-05-17

**Authors:** Martina Stekrova, Narendra Kumar, Päivi Mäki-Arvela, Oleg V. Ardashov, Konstantin P. Volcho, Nariman F. Salakhutdinov, Dmitry Yu. Murzin

**Affiliations:** 1 Laboratory of Industrial Chemistry and Reaction Engineering, Åbo Akademi University, Biskopsgatan 8, Turku 20500, Finland; E-Mails: stekrovm@vscht.cz (M.S.); nkumar@abo.fi (N.K.); pmakiarv@abo.fi (P.M.-A.); 2 Department of Organic Technology, Institute of Chemical Technology Prague, Technická 5, Prague 16000, Czech Republic; 3 VUAnCH Litvínov, a. s., Revolucni 1524, Ústí nad Labem 400 01, Czech Republic; 4 N. N. Vorozhtsov Novosibirsk Institute of Organic Chemistry, Siberian Branch, Russian Academy of Sciences, Lavrentyev Ave. 9, Novosibirsk 630090, Russia; E-Mails: ardashov@nioch.nsc.ru (O.V.A.); volcho@nioch.nsc.ru (K.P.V); anvar@nioch.nsc.ru (N.F.S.)

**Keywords:** cerium, Ce–Si–MCM-41, Ce–H–MCM-41, Ce–Si–SBA-15, isomerization, α-pinene oxide, *trans*-carveol

## Abstract

Ce-modified mesoporous silica materials MCM-41 and SBA-15, namely 32 wt % Ce–Si–MCM-41, 16 wt % Ce–H–MCM-41 and 20 wt % Ce–Si–SBA-15, were prepared, characterized and studied in the selective preparation of *trans*-carveol by α-pinene oxide isomerization. The characterizations of these catalysts were performed using scanning electron microscopy, X-ray photoelectron spectroscopy, nitrogen adsorption and FTIR pyridine adsorption. Selective preparation of *trans*-carveol was carried out in the liquid phase in a batch reactor. The activity and the selectivity of catalyst were observed to be influenced by their acidity, basicity and morphology of the mesoporous materials. The formation of *trans*-carveol is moreover strongly influenced by the basicity of the used solvent and in order to achieve high yields of this desired alcohol it is necessary to use polar basic solvent.

## 1. Introduction

Isomerization reactions of the terpene oxides have become a very interesting topic in the recent years [1,2]. The products of these reactions possess various utilizations as chemical specialties or useful intermediates for production of drugs, vitamins and fragrances.

**Figure 1:**
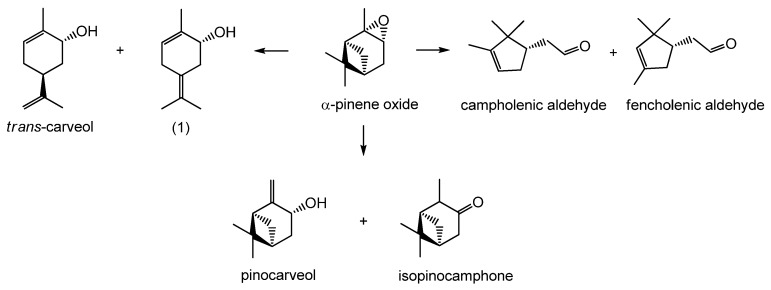
Reaction schemes of α-pinene oxide isomerization to *trans*-carveol, (1) 2-methyl-5-(propan-2-ylidene)cyclohex-2-enol, campholenic aldehyde, fencholenic aldehyde, pinocarveol and isopinocamphone.

α-Pinene oxide is one of these very reactive substrates, which isomerizes rapidly in the presence of acids, thereby forming many products. The industrially most desired products of α-pinene oxide isomerization are *trans*-carveol and campholenic aldehyde (Figure 1) because they are highly valuable ingredients for the production of flavors. *Trans*-carveol has been additionally found to exhibit chemoprevention of mammary carcinogenesis [3,4]. Compared to selective preparation of campholenic aldehyde the selective synthesis of *trans*-carveol has been described only in a few publications. 

The high selectivity, 73% to *trans*-carveol at 98% conversion, was achieved using cerium and tin supported catalysts and a polar basic solvent: dimethylacetamide [5]. The high yield of *trans*-carveol was also obtained using molecularly imprinted polymers as a protic catalyst with *N*,*N*-dimethylformamide as a solvent [6].

The main focus of this research was on testing ceria supported by various ordered mesoporous silica materials for selective preparation of *trans*-carveol. Activity and selectivity of these catalysts were correlated with their physico-chemical properties; mainly with their acidity, structure of mesoporous materials and loaded amount of ceria. Ordered mesoporous silica materials have attracted much attention in the recent years, because of their potential applications in catalysis. The most common types of mesoporous materials are MCM-41 and SBA-15.

Several ceria supported on mesoporous materials MCM-41 and SBA-15 catalysts were prepared in the current research, namely 32 wt % Ce–Si–MCM-41, 16 wt % Ce–H–MCM-41 and 20 wt % Ce–Si–SBA-15. The basic properties of these catalysts were discussed previously [7,8]. This is the first time when these catalysts were tested for a reaction, typically catalyzed by acids.

Cerium ions incorporated in on MCM-41 has been tested previously for various reactions. Ce–MCM-41 was applied for ozonation of *p*-chlorobenzoic acid in an aqueous solution and significantly improving the oxidation rate [9,10]. Ce–MCM-41 catalyst was found to be active in oxathiacetalization, alkylation and epoxidation catalyzed processes [11]. The use of Ce–MCM-41 was reported for the synthesis of unsymmetric biaryls via oxidative cross coupling reactions [12]. Ce–MCM-41 also exhibited high catalytic activity and good selectivity in the liquid phase oxidation of cyclohexane using aqueous hydrogen peroxide as an oxidant and acetic acid as a solvent [13]. Ce–MCM-41 was studied as a selective catalyst for acylation and alkylation reactions. It was used for acylation of alcohols, thiols, phenols and amines displaying good activity and selectivity. For example, Ce–MCM-41 was described as an effective catalyst for the acylation of cholesterol, ergosterol and β-sitosterol [14].

Ce–SBA-15 has been tested in the previous studies mainly for oxidation reactions. Ce–SBA-15 has been described as an effective catalyst in oxidative cleavage of cyclohexene to adipic acid using aqueous hydrogen peroxide as an oxidant [15]. Ce–silica mesoporous SBA-15-type material were applied for the cyclohexanol and cyclohexene oxidation with hydrogen peroxide [16].

## 2. Experimental Section 

### 2.1. Catalyst Synthesis 

Ceria-modified MCM-41 and SBA-15 mesoporous materials were prepared and investigated in the present study. 

32 wt % Ce–Si–MCM-41 and 16 wt % Ce–H–MCM-41 were prepared by evaporation impregnation. Mesoporous materials were synthesized in the forms of Na–Si–MCM-41 and Na–Al–MCM-41 using a Parr autoclave (300 mL) as mentioned in [17] with few modifications [7]. After synthesis of MCM-41, it was filtered, washed with distilled water, dried overnight at 100 °C and calcined. Proton form H–MCM-41 was prepared by ion-exchange with ammonium chloride, followed by washing with distilled water, drying and calcination at 450 °C. Ceria modification of Na–Si–MCM-41 and of H–MCM-41 mesoporous materials was carried out using evaporation impregnation method and a rotator evaporator. Cerium nitrate was used as a cerium precursor. After modification the catalysts were dried at 100 °C and calcined at 550 °C.

Ce–Si–SBA-15 was prepared by deposition-precipitation method. Si–SBA-15 mesoporous material was synthesized as mentioned in the reference [18,19]. The solid product was filtered, washed with deionized water, dried overnight at 90 °C and calcined at 550 °C in order to remove the organic template. Si–SBA-15 mesoporous material modified by CeO_2_ was prepared by deposition-precipitation method [8]. The prepared and calcined Si–SBA-15 was dispersed in aqueous solution of urea. The appropriate amount of precursor cerium nitrate was dissolved in deionized water and added to the urea and support suspension. The suspension was stirred for 5 h at 70 °C. In the next step, aqueous ammonia was added into the suspension in order to increase pH to 9 and stirring was continued for an hour. The final catalyst was filtered, washed by deionized water, dried overnight at 100 °C and calcined at 600 °C. 

### 2.2. Catalyst Characterization 

The catalysts were characterized using SEM, nitrogen adsorption, FTIR spectroscopy with pyridine as a probe molecule and XPS.

Morphological studies were performed by scanning electron microscopy. The scanning electron microscope (Zeiss Leo Gemini 1530) was used for determining the crystal morphology of the ceria-supported mesoporous catalysts.

The specific surface area of mesoporous materials per se and of ceria modified catalysts was determined by nitrogen adsorption using Sorptometer 1900 (Carlo Erba instruments). The samples were outgassed at 150 °C for 3 h before each measurement. The BET equation was used for calculation of the specific surface area of catalysts.

The acidity of the mesoporous materials and of ceria supported catalysts was measured by infrared spectroscopy (ATI Mattson FTIR) using pyridine (≥99.5%) as a probe molecule for qualitative and quantitative determination of both Brønsted and Lewis acid sites. The samples were pressed into thin pellets (10–25 mg). The pellets were pretreated at 450 °C before the measurement. Pyridine was first adsorbed for 30 min at 100 °C and then desorbed by evacuation at different temperatures. Three different temperatures were used for desorption of pyridine, defined as 250–350 °C as weak, medium and strong sites, 350–450 °C as medium and strong sites as well as pyridine which stays adsorbed after desorption at 450 °C as strong sites [20]. The amount of Brønsted and Lewis acid sites were calculated from the intensities of the corresponding spectral bands, 1545 cm^−1^ and 1450 cm^−1^ respectively, using the molar extinction parameters previously reported by Emeis [21]. The catalysts weights were taken into account in the calculations.

The photoemission spectra were measured using a Perkin-Elmer PHI 5400 spectrometer with a monochromatized Al Kα X-ray source that was operated at 14 kV, 300 W. The analyzer pass energy was 18 eV and the energy step was 0.1 eV. 

### 2.3. Catalytic Experiments 

The isomerization of α-pinene oxide (97%, Aldrich) over Ce-modified catalysts was carried out in a laboratory scale in the liquid phase using a batch-wise glass reactor. In a typical experiment the initial concentration of epoxide and the catalyst mass were 0.02 mol/L and 75 mg, respectively. The kinetic experiments were performed under the following conditions to avoid external mass transfer limitation: the catalysts particle size below 90 µm and the stirring speed of 390 rpm. The catalyst was activated in the reactor at 250 °C under an inert argon atmosphere for 30 min before the reaction. The polar solvents with various basicity were tested for α-pinene oxide isomerization (V_L_ = 100 mL). The reaction temperature was 140 °C, or the reaction was performed under reflux when the boiling point of the used solvent was lower than 140 °C. The samples were taken at different time intervals and analyzed by GC using a DB-Petro column with a capillary of 100 m × 250 µm × 0.50 µm nominal (Agilent 128–1056) and with a FID detector. 

The products were identified and confirmed by GC-MS using an Agilent 7890A gas chromatograph equipped with a quadrupole mass spectrometer Agilent 5975C as a detector. Quartz column HP-5MS (copolymer 5%–diphenyl–95%–dimethylsiloxane) of length 30 m, internal diameter 0.25 mm and stationary phase film thickness 0.25 µm was used for the analysis.

2-Methyl-5-(propan-2-ylidene)cyclohex-2-enol (1) was isolated from the reaction mixture using column chromatography on SiO_2_. ^1^H NMR spectrum (AV-300, Bruker) of the compound coincided with respective spectrum reported in literature [5].

## 3. Results and Discussion

### 3.1. Catalyst Characterization

#### 3.1.1. Morphological Studies by Scanning Electron Microscopy 

**Figure 2:**
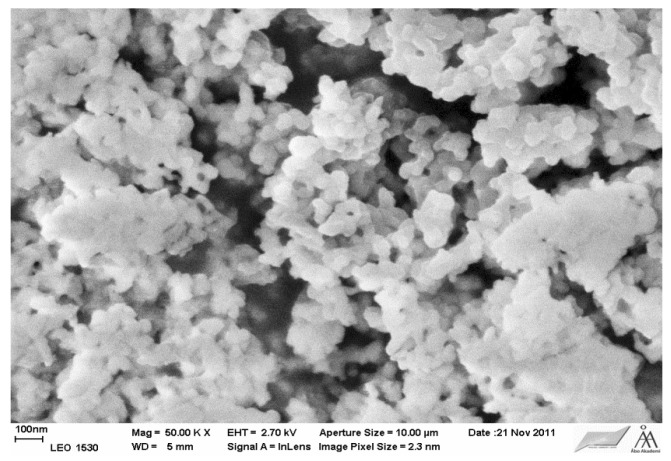
Scanning electron micrograph (SEM) of 16 wt % Ce–H–MCM-41.

**Figure 3:**
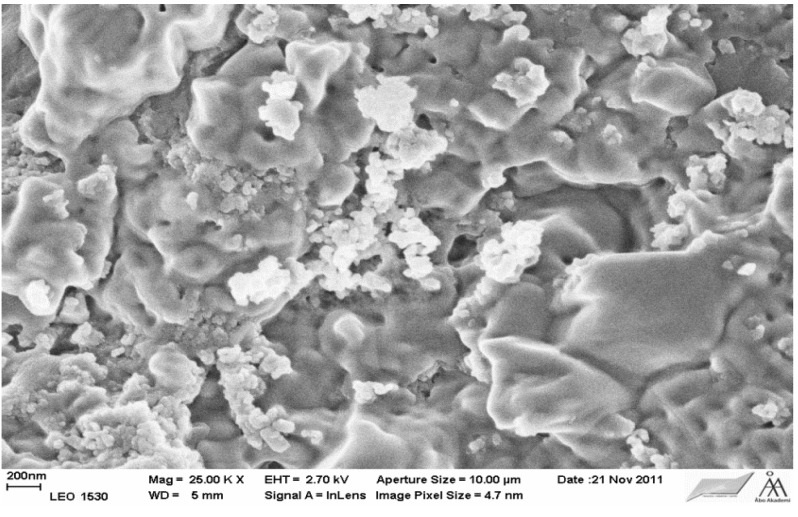
SEM of 20 wt % Ce–Si–MCM-41.

The morphology (shape and size) of the Ce–Si–MCM-41 and Ce–H–MCM-41 catalysts was studied by scanning electron microscopy (Figure 2 and Figure 3). The phases typical for MCM-41 material were observed in the both cases. MCM-41 material exhibited thin walls and about 4 nm. On the other hand, for 32 wt % Ce–Si–MCM-41 sample also a morphologically non-typical phase for Si–MCM-41. It is very probable that the high loading amount of ceria (32 wt %) caused a partial collapse of the structure of Si–MCM-41. 

The SEM and TEM images of 20 wt % Ce–Si–SBA-15 were obtained and published previously [8]. SEM images showed long tube shaped particles typical for SBA-15 mesoporous material. TEM images revealed typical U-shaped pores in siliceous SBA-15 with relatively thick walls and larger pores than those of H–MCM-41 [8].

#### 3.1.2. X-ray Photoelectron Spectroscopy 

The X-ray photoelectron spectroscopy of ceria catalysts demonstrated that the highest amount of Ce^3+^ (66%) was exhibited by Ce–Si–MCM-41 mesoporous material with the highest amount of ceria (32 wt %). Ce^4+^ (34%) was also present in this catalyst. The amounts of Ce^3+^ and Ce^4+^ in Ce–H–MCM-41 are 46% and 54%, respectively [7]. Only Ce^4+^ was observed in Ce–Si–SBA-15 [8]. The presence of only Ce^4+^ in SBA-15 may be attributed to the thicker pores walls and larger pore size of SBA-15 than MCM-41 mesoporous material.

#### 3.1.3. Specific Surface Areas of the Catalysts 

The specific surface areas of the Ce-modified mesoporous materials were determined by nitrogen adsorption and calculated by BET method. The specific surface areas decreased after the ceria loading due to the blocking of some pores by the metal inserted. The lowest specific surface area was determined for 32 wt % Ce–Si–MCM-41 being 384 m^2^/g due to the high loading of ceria and a probable partial distortion of the mesoporous phase. High specific areas were determined for 16 wt % Ce–H–MCM-41 and 20 wt % Ce–Si–SBA-15 catalysts being 852 m^2^/g and 598 m^2^/g, respectively [7,8]. 

#### 3.1.4. Acid Site Concentrations of the Catalysts Measured by FTIR 

The amount of Brønsted and Lewis acid sites were determined by pyridine adsorption with FTIR (Table 1). The highest concentrations of Lewis and Brønsted acid sites exhibited Ce–Si–MCM-41 catalyst with the highest loading of ceria. The corresponding non-modified mesoporous material, Na–Si–MCM-41, exhibited quite low concentration of Lewis acid sites.

**Table 1:** Brønsted and Lewis acidities of Ce modified catalysts.

Catalysts	Brønsted acidity (µmol/g)			Lewis acidity (µmol/g)			amount of basic sites (mmol/g_cat_) [7,8]
	250 °C (weak, medium and strong)	350 °C (medium and strong)	450 °C (strong)	250 °C (weak, medium and strong)	350 °C (medium and strong)	450 °C (strong)	–
**Na–Si–MCM-41**	0	0	0	36	10	3	–
**32 wt % Ce–Si–MCM-41**	**80**	**49**	**10**	**67**	**12**	**9**	**2.53**
**H–MCM-41**	39	21	5	32	18	7	–
**16 wt % Ce–H–MCM-41**	**52**	**9**	**1**	**31**	**3**	**0**	**16.25**
**Si–SBA-15**	2	2	1	31	15	4	–
**20 wt % Ce–Si–SBA-15**	**17**	**1**	**1**	**50**	**17**	**6**	**4.4**

The increase of the amount of only weak Brønsted acid sites was observed after loading of ceria on mesoporous H–MCM-41 as well on SBA-15, whereas the amount of strong acid sites decreased. This trend of increasing of Brønsted acidity after loading Ce on silicate or alumosilicate materials has been already described previously [22,23]. The amount of weak Lewis acid sites increases in case of loading ceria to mesoporous SBA-15. The amount of medium and strong Lewis acid sites in case of Ce–SBA-15 is almost the same in comparison with pure SBA-15. The amount of medium and strong Brønsted as well as Lewis acid sites decreased after introducing of ceria on mesoporous H–MCM-41. The amount of basic sites were measured by CO_2_ desorption in [7,8] and reported in Table 1 as well. The highest basicity was measured for 16 wt % Ce–H–MCM-41.

### 3.2. Results from the Catalytic Tests

Three different ceria modified ordered mesoporous silica materials with different physico-chemical properties were studied in α-pinene oxide isomerization. The activities and selectivities of all catalysts were mainly correlated with acid and base properties of the catalysts as well as their structures. 

In addition, the influence of the basicity of the used solvents was studied.

#### 3.2.1. Isomerization of α-pinene Oxide: Effect of Catalyst Properties

The results of the catalytic experiments focused on the preparation of *trans*-carveol by α-pinene oxide isomerization over ceria modified mesoporous materials are shown in Table 2. 

**Table 2:** Initial reaction rate within the 10 min from the beginning of isomerization reaction, conversion of α-pinene oxide and selectivities to the desired *trans*-carveol and to the by-product campholenic aldehyde at 10% conversion of α-pinene oxide.

Catalyst	Initial reaction rate (mmol/min·L·g_cat_)	Initial reaction rate (mmol/min·L·g_cat_)	Conversion of APO after 3 h (%)	Selectivity to TCV at 10% conversion of APO (%)	Selectivity to CA at 10% conversion of APO (%)
32 wt % Ce–Si–MCM-41	2.9	9.1	100	35	38
16 wt % Ce–H–MCM-41	1.1	6.9	33	14	59
20 wt % Ce–Si–SBA-15	0.4	2	12	27	40

Note: Reaction conditions: 140 °C, DMA; APO = α-pinene oxide, TCV = *trans*-carveol, CA = campholenic alcohol.

The highest initial reaction rate within 10 minutes from the beginning of the reaction was achieved using Ce–Si–MCM-41. This catalyst has the highest loading of ceria being 32 wt % and the highest amount of Lewis and Brønsted acid sites. Furthermore, this catalyst was the least basic of all the three studied catalysts exhibiting the basicity of 2.53 mmol/g_cat_ [7,8]. The most basic catalyst, Ce–H–MCM-41 with the basicity of 16.53 mmol/g_cat._ was the second most active catalyst. The total conversion of α-pinene oxide was achieved using 32 wt % Ce–Si–MCM-41 catalyst within 3 h from the beginning of the reaction. As a comparison 98% conversion of α-pinene oxide was achieved in 8 h over Ce/SiO_2_ in [4]. 

**Figure 4:**
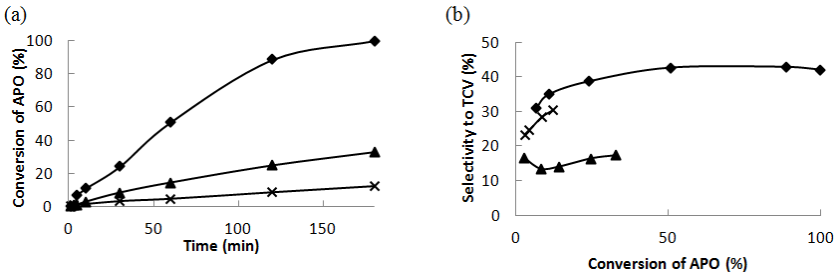
(**a**) The conversion of α-pinene oxide at 140 °C in dimethylacetamide as a function of reaction time and (**b**) the selectivity to *trans*-carveol as a function of the conversion of α-pinene oxide over 32 wt % Ce–Si–MCM-41 (♦), 16 wt % Ce–H–MCM-41 (▲) and 20 wt % Ce–Si–SBA-15 (X).

The conversion of α-pinene oxide as a function of reaction time over Ce supported catalysts is depicted in Figure 4a. Low conversions of α-pinene oxide were achieved using the two other less acidic catalysts, 16 wt % Ce–H–MCM-41 and 20 wt % Ce–Si–SBA-15 being 33% and only 12%, respectively, after three hours of reaction. Thus, it can be concluded that the activity of catalyst decreases by decreasing the amount of Brønsted acid sites.

Selectivity to *trans*-carveol and to campholenic aldehyde seems to be influenced by the acid properties of catalysts. The lowest selectivity to *trans*-carveol was achieved using 16 wt % Ce–H–MCM-41, catalyst exhibiting the lowest amount of Lewis acid sites. The highest selectivity to this desired alcohol was achieved using 32 wt % Ce–Si–MCM-41 with the highest amount of Lewis acid sites and with the lowest basicity. By decreasing the amount of Lewis acid sites, the selectivity to *trans*-carveol decreased and at the same time the selectivity to campholenic aldehyde increased. It is an unexpected result because just homogeneous Lewis acids are the ordinary catalysts for campholenic aldehyde synthesis from the epoxide [24]. High selectivity to *trans*-carveol, about 73% was reported over Ce/SiO_2_ in DMA as a solvent at 140 °C in [4], but neither catalyst acidity nor basicity were not reported in [4]. The product distribution varied over different ceria catalysts. The selectivity to the desired *trans*-carveol as a function of α-pinene oxide conversion is depicted in Figure 4b. 32 wt % Ce–Si–MCM-41 catalyst exhibited the highest selectivity to *trans*-carveol at α-pinene oxide conversion of 89%. The selectivity to *trans*-carveol over 32 wt % Ce–Si–MCM-41 decreased to 41% at the total conversion of α-pinene oxide.

**Figure 5:**
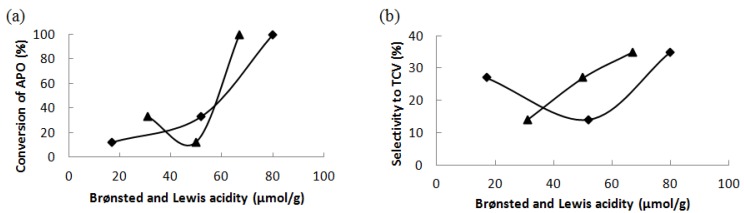
(**a**) The conversion of α-pinene oxide after 180 min and (**b**) the selectivity to *trans*-carveol at 10% conversion as the functions of the concentration of Brønsted (♦) and Lewis (▲) acid sites at 140 °C in dimethylacetamide as a solvent.

The results show that the main parameters influencing the activity and selectivity are the acid and base properties of the catalysts, structure and amount of Ce. Figure 5a,b show that the activity increases with the amount of all (weak, medium and strong) Brønsted acid sites. The dependence of the conversion of α-pinene oxide on the amount of all Lewis acid sites exhibits the minimum. On the other hand, the selectivity of catalysts to *trans*-carveol increases with increasing amount of Lewis acid sites. The minimum in the dependence of the selectivity on the amount of Brønsted acid sites can be observed in Figure 5b. Furthermore, the selectivity towards campholenic aldehyde increased with increasing basicity of the catalyst, whereas an opposite trend was observed for selective formation of *trans*-carveol, for which the highest selectivity was achieved with the least basic catalyst, Ce–Si–MCM-41. 

**Figure 6:**
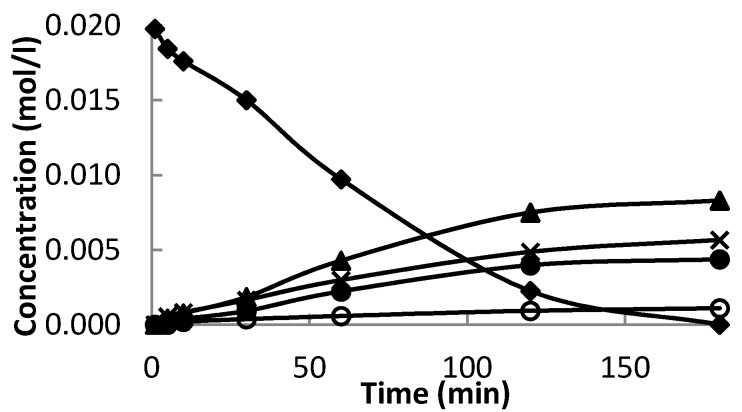
Kinetics in the α-pinene oxide isomerization over 32 wt % Ce–Si–MCM-41 at 140 °C, using dimethylacetamide as a solvent. Symbols: α-pinene oxide (♦), *trans*-carveol (▲), campholenic aldehyde (X), fencholenic aldehyde (o) and 2-methyl-5-(propan-2-ylidene)cyclohex-2-enol (●).

Minor often encountered products are fencholenic aldehyde and 2-methyl-5-(propan-2-ylidene)cyclohex-2-enol. Figure 6 displays concentration of the reactant and all products over the most active and selective catalyst 32 wt % Ce–Si–MCM-41 using *N*,*N*-dimethylacetamide as a solvent. The formation of 2-methyl-5-(propan-2-ylidene)cyclohex-2-enol was previously observed in the only case as a minor product of α-pinene oxide isomerization in the presence of molecularly imprinted polymer in DMF [5].

The initial reaction rate of *trans*-carveol formation was slightly lower than initial reaction rate of campholenic aldehyde formation being 1.1 mmol/(min·L·g_cat_) and 1.5 mmol/(min·L·g_cat_), respectively, over 32 wt % Ce–Si–MCM-41. Higher *trans*-carveol formation rates than of campholenic aldehyde formation could be observed already after 10 min of reaction with the highest ratio equal to 1.5 after 120 min.

**Figure 7:**
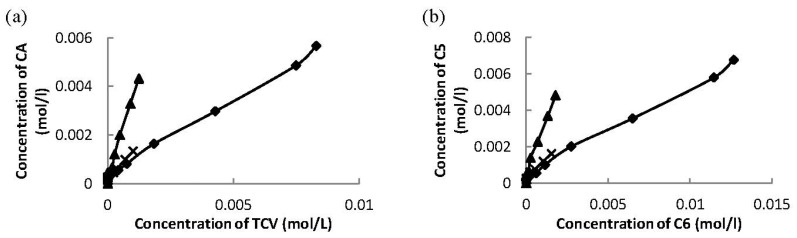
**(a)** Concentration of campholenic aldehyde versus concentration of *trans*-carveol and **(b)** concentration of C5 products (sum of concentration of campholenic aldehyde and fencholenic aldehyde) versus concentration of C6 products (sum of concentration of *trans*-carveol and 2-methyl-5-(propan-2-ylidene)cyclohex-2-enol) over 32 wt % Ce–Si–MCM-41 (♦), 16 wt % Ce–H–MCM-41 (▲) and 20 wt % Ce–Si–SBA-15 (X).

The ratio between the concentrations *trans-*carveol and campholenic aldehyde increased during the reaction over these three ceria supported on mesoporous materials (Figure 7a). The same trend was observed for the concentrations of the products with 5-member carbon-ring (campholenic aldehyde and fencholenic aldehyde) and products with 6-member carbon-ring (*trans*-carveol and 2-methyl-5-(propan-2-ylidene)cyclohex-2-enol) (Figure 7b) indicating parallel formation of the two C5 products, campholenic and fencholenic aldehyde, which was also the case for C6 products, *trans*-carveol and 2-methyl-5-(propan-2-ylidene)cyclohex-2-enol.

#### 3.2.2. Isomerization of α-pinene Oxide: Effect of the Basicity of Solvents

The reaction is strongly influenced by the basicity of the solvent. The optimal solvent for campholenic aldehyde preparation is toluene [2]—a solvent with no Lewis basicity. A polar basic solvent is necessary to be used for the selective preparation of *trans*-carveol [4]. Activity and selectivity of 32 wt % Ce–Si–MCM-41 was evaluated using besides *N*,*N*-dimethylacetamide (DMA) the following solvents: acetonitrile, tetrahydrofuran (THF), pentan-2-ol and *N*-methylpyrrolidone (NMP).

A comparison of solvents basicity of solvents is shown in Table 3. The Lewis basicity of solvents was measured using various methods and equations [25,26]. The solvent Lewis basicity with particular consideration and extension of Kamlet-Taft scale (B_KT_) were reported in [25]. B_KT_ values for 70 solvents were measured and additionally combined in [26]. 

**Table 3:** Basicity characteristics of solvents according [25,26].

**Solvent**	**Reported B_KT_ values**	
	[25]	[26]
Toluene	–	0.00
Acetonitrile	–	0.23
Tetrahydrofuran	0.55	0.47
*n-Pentanol*	–	0.72
*N*,*N*-dimethylacetamide	0.73	–
*N*-methylpyrrolidone	0.75	–

Note: No data available for pentan-2-ol.

**Figure 8:**
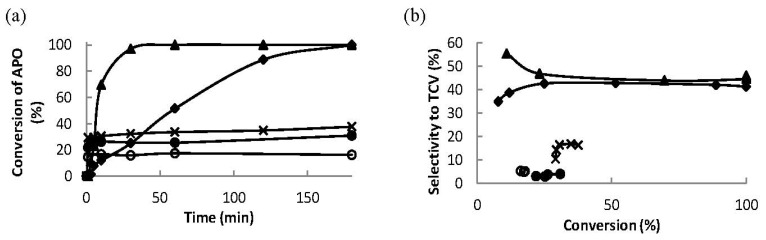
(**a**) Conversion of α-pinene oxide as a function of reaction time and (**b**) selectivity to *trans*-carveol as a function of conversion of α-pinene oxide at 140 °C over 32 wt % Ce–Si–MCM-41 using various solvents: DMA (♦), NMP (▲), pentan-2-ol (X), THF (o) and acetonitrile (●).

The conversion of α-pinene oxide as a function of reaction time over the most active Ce–MCM-41 catalyst using solvents with various basicities is depicted in Figure 8a. The total conversion of α-pinene oxide was achieved using *N*-methylpyrrolidone within 30 min from the beginning of the reaction as well as using dimethylacetamide as a solvent within 180 min. 

Catalyst deactivation occurred in all solvents except *N*-methylpyrrolidone and dimethylacetamide (Figure 8a). The conversion achieved after 3 h increased with increasing basicity of the solvent (Table 4) as follows: 2-pentanol (38%) < dimethylacetamide (100%) = *N*-methylpyrrolidone (100%) thus indicating that the catalyst activity remained high with the solvents exhibiting the highest basicity. On the other hand, the dielectric coefficient of the solvent is a measure of polarity is for acetonitrile 37.5, which is about the same as those for dimethylacetamide (37.8) and *N*-methylpyrrolidone (32.2). 

**Table 4:** Initial reaction rate in isomerization of α-pinene oxide using various solvents.

Solvent	Initial reaction rate (after 10 min)	Conversion of APO after 3 h (%)
	(mmol/ min·L·g_cat_)	
Acetonitrile *	6.4	31
Tetrahydrofuran *	3.9	16
Pentan-2-ol ***	8	38
**Dimethylacetamide**	2.9	100
***N*-methylpyrrolidone**	18	100

Note: Reaction conditions: 32 wt % Ce–Si–MCM-41, 140 °C; * 82 °C, ** 66 °C, *** 119 °C; APO = α-pinene oxide.

The selectivity to the desired *trans*-carveol is influenced by the basicity of used solvent and by the reaction temperature. The selectivities to the desired alcohol as a function of conversion of α-pinene oxide are depicted in Figure 8b. The highest selectivity to the desired alcohol was achieved by using *N*-methylpyrrolidone being 55% at 10% conversion of α-pinene oxide and 46% at the total conversion of α-pinene oxide. The selectivity to *trans*-carveol was 41% at complete conversion of α-pinene oxide when using dimethylacetamide as a solvent. The highest selectivity towards formation of *trans*-carveol in the present study is maximally 55% at total conversion of α-pinene in *N*-methylpyrrolidone. It has been reported in [4] that 73% selectivity at 98% conversion towards *trans*-carveol was achieved in dimethylacetamide as a solvent at 140 °C over Ce/SiO_2_ catalyst, in which the ceria was prepared using CeCl_3_ as a precursor. It should, however, be pointed out here, that their results in [4] were not correlated with acidity or basicity of the utilized catalysts. 

The selectivity to *trans*-carveol increased with increasing basicity of the solvent, at the same time the selectivity to campholenic aldehyde decreased (Table 5). The same trend was observed in the formation of C6 (6-member carbon-ring products: *trans*-carveol and 2-methyl-5-(propan-2-ylidene)cyclohex-2-enol) and C5 (5-member carbon-ring products: campholenic aldehyde and fencholenic aldehyde) products. 

**Table 5:** The selectivities to *trans*-carveol, campholenic aldehyde, and to sum of C6 and C5 products at 30% conversion (at 100%, respectively) of α-pinene oxide over 32 wt % Ce–Si–MCM-41.

Solvent	Selectivities (%) at 30% conversion of APO ^a^ (at 100% conversion)			
	TCV	CA	C6	C5
Acetonitrile *	4	48	9	54
Tetrahydrofuran **	5	47	5	56
Pentan-2-ol ***	16	44	22	52
Dimethylacetamide	39 (41) ^a^	34 (28) ^a^	58 (65) ^a^	42 (34) ^a^
*N*-methylpyrrolidone	46 (46) ^a^	31 (26) ^a^	68 (68) ^a^	32 (32) ^a^

Note: Reaction conditions: 140 °C; * 82 °C, ** 66 °C, *** 119 °C; ** selectivities at 16% conversion of APO; APO = α-pinene oxide, TCV = *trans*-carveol, CA = campholenic alcohol; C6 = *trans*-carveol and 2-methyl-5-(propan-2-ylidene)cyclohex-2-enol; C5 = campholenic and fencholenic aldehyde; ^a^: at 100% conversion of α-pinene oxide.

**Figure 9:**
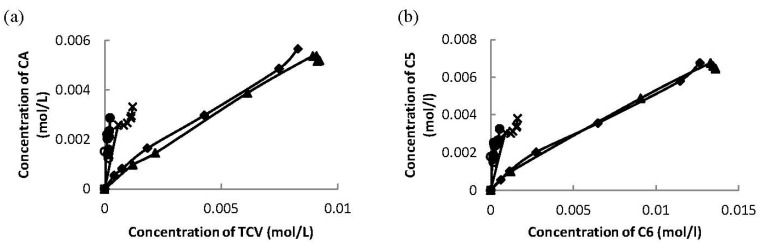
(**a**) The concentrations of campholenic aldehyde versus concentrations of *trans*-carveol and (**b**) concentrations of C5 products (sum of concentration of campholenic aldehyde and fencholenic aldehyde) versus concentrations of C6 products (sum of concentration of *trans*-carveol and 2-methyl-5-(propan-2-ylidene)cyclohex-2-enol) over 32 wt % Ce–Si–MCM-41 using various solvents: DMA (♦), NMP (▲), pentan-2-ol (X), THF (o) and acetonitrile (●).

The ratio between *trans*-carveol and campholenic aldehyde during the reaction over tested solvents was constant, except for 2-pentanol (Figure 9a), indicating parallel formation of these products in *N*-methylpyrrolidone and dimethylacetamide. The same trend was observed for the formation with 5-member carbon-ring (campholenic aldehyde and fencholenic aldehyde) and 6-member carbon-ring (*trans*-carveol and 2-methyl-5-(propan-2-ylidene)cyclohex-2-enol) products (Figure 9b) analogously as in case of using different Ce-catalysts.

**Figure 10:**
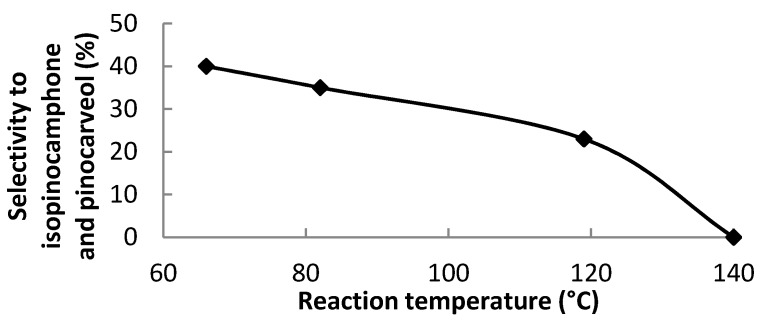
The sum of selectivities to isopinocamphone and pinocarveol as a function of the reaction temperature.

Minor products, often encountered besides the above mentioned fencholenic aldehyde and 2-methyl-5-(propan-2-ylidene)cyclohex-2-enol, are isopinocamphone and pinocarveol, e.g., the products with only epoxide ring opened (Figure 1a). These products are formed using solvents with lower basicity. The selectivity to isopinocamphone and pinocarveol is given in Table 6. The highest selectivity to these undesired products was obtained using tetrahydrofuran. It may be explained by the lowest reaction temperature (66 °C). The sum of selectivities to these non-splitting products as a function of the reaction temperature is displayed in Figure 10. The selectivity to these products decreases with the increasing reaction temperature. It should be noted that relatively selective isomerization of α-pinene oxide into the compounds with pinane framework is not a simple task and usually require specific catalysts such as Au/TiO_2_ [27] or high temperature thermolysis [28].

**Table 6:** The selectivities to undesired by-products isopinocamphone and pinocarveol at 30% conversion of α-pinene oxide over 32 wt % Ce–Si–MCM-41.

Solvent	Selectivities at 30% conversion of APO ^a^ (at 100% conversion)	
	Isopinocamphone	Pinocarveol
Acetonitrile *	25	10
Tetrahydrofuran **	28	12
Pentan-2-ol ***	15	8
Dimethylacetamide	0 (0) ^a^	0 (0) ^a^
*N*-methylpyrrolidone	0 (0) ^a^	0 (0) ^a^

Note: Reaction conditions: 140 °C; * 82 °C, ** 66 °C, *** 119 °C; ** selectivities at 16% conversion of APO; ^a^: at 100% conversion of α-pinene oxide.

## 4. Conclusions 

Ce-modified mesoporous materials MCM-41 and SBA-15, namely 32 wt % Ce–Si–MCM-41, 16 wt % Ce–H–MCM-41 and 20 wt % Ce–Si–SBA-15, were prepared, characterized and studied in the selective preparation of *trans*-carveol by α-pinene oxide isomerization. 

Isomerization of α-pinene oxide at 140 °C using *N*,*N*-dimethylacetamide as a solvent shows that the main properties influencing the activity and the selectivity are the acid and base properties of the catalysts. The activity increases by increasing amount of Brønsted acid sites, whereas the least basic catalyst, namely Ce–Si–MCM-41, was also the most active one. The selectivity to *trans*-carveol increases by increasing the amount of Lewis acid sites. The highest concentration of Brønsted and Lewis acid sites was observed for 32 wt % Ce–Si–MCM-41, which was also the least basic of all the three studied catalysts. The total conversion of α-pinene oxide was achieved using this catalyst within 180 min giving selectivity to desired alcohol equal 41%.

Isomerization of α-pinene oxide is also strongly influenced by the basicity of the used solvent. The polar basic solvent is necessary to be used for the selective preparation of *trans*-carveol. The activity and the selectivity of 32 wt % Ce–Si–MCM-41 was evaluated using the following solvents: *N*,*N*-dimethylacetamide, acetonitrile, tetrahydrofuran, pentan-2-ol and *N*-methylpyrrolidone. Both the activity and selectivity to *trans*-carveol increases by increasing basicity of the solvent, at the same time the selectivity to campholenic aldehyde decreases. The highest selectivity to desired alcohol was achieved using *N*-methylpyrrolidone as a solvent being 46% at total conversion of α-pinene oxide. 
